# Maturity-specific trajectories of physical fitness in talented youth athletes: a longitudinal study of Japanese local talent identification and development programme

**DOI:** 10.3389/fspor.2026.1821744

**Published:** 2026-05-26

**Authors:** Masahiro Hagiwara, Taisuke Kinugasa, Miki Haramura, Takehira Nakao, Kazuto Teshima, Shuhei Yamashita, Katsuyoshi Shirai

**Affiliations:** 1 Japan Institute of Sports Sciences, Japan High Performance Sport Center, Japan Sport Council, Tokyo, Japan; 2 The Center for Liberal Arts, Meiji Gakuin University, Kanagawa, Japan; 3 Faculty of Human Science, Kyushu Sangyo University, Fukuoka, Japan; 4 Fukuoka Prefecture Institute of Sports Science and Information, Fukuoka Prefecture Sports Council, Fukuoka, Japan

**Keywords:** adolescent awkwardness, anthropometry, athletic potential, peak height velocity, performance stability, trainability

## Abstract

**Purpose:**

Despite the global emphasis on talent identification and development (TID), longitudinal evidence on physical development in Asian youth remains scarce. Using Japan's local TID (L-TID) multi-sport matching model, this study examined longitudinal trajectories of physical fitness in talented Japanese youth athletes categorised by maturity status.

**Methods:**

Data were analysed from 96 participants (43 males, 53 females) in a Fukuoka L-TID programme, followed from Grade 4 to Grade 9. Somatic maturity (Early, Average, or Late) was based on the timing of peak height velocity (PHV). Measurements included height, body mass, 25-m sprint, standing broad jump (SBJ), reaction time (RT), and 20-m shuttle run (SR).

**Results:**

In males, improvements in SBJ and sprint speed synchronised with the PHV period (*p* < 0.05), supported by medium-to-large interaction effects. Conversely, females demonstrated a significant post-PHV decline in SR (*p* < 0.05), suggesting a transient physiological constraint. RT improved independently of maturation across both sexes (*p* < 0.05), reaching a plateau between Grades 8 and 9. Correlation analysis revealed substantial performance ranking instability between programme entry and exit, particularly within the Average maturity group (*r* = 0.09–0.22).

**Conclusion:**

Biological maturation acts as an accelerator for males but a constraint for females. Observed ranking instability suggests proximity to PHV disrupts motor coordination, requiring neuromuscular re-integration. Therefore, these findings suggest that early-stage TID programmes could consider integrating neurocognitive development to establish a stable foundation before the onset of maturation-related physical fluctuations.

## 1 Introduction

In the high-performance sport environment, improving and sustaining international competitiveness has become a major national priority. Consequently, effective talent identification and development (TID) programmes are widely recognised as key components of successful sport systems (1–3). Over recent decades, many countries have invested in structured athlete development pathways to identify and support athletic potential from an early age (4, 5). Contemporary frameworks increasingly adopt multidimensional perspectives incorporating physical, psychological, and sociological factors, while prioritising long-term planning and flexibility across different sports (3, 4, 6). In Japan, optimising athlete development pathways is a national priority under the “Third Basic Sport Plan” and the “Sustainable Plan for improving International Competitiveness” (7, 8). These initiatives aim to strengthen international competitiveness through targeted resource allocation and TID at the local (prefectural) level.

There is growing interest among researchers in athlete development models based on scientific evidence, rather than short-term evaluations of current performance (9, 10). In this context, long-term development strategies highlight the benefits of multi-sport engagement and “sport sampling”, which may foster broad motor skill acquisition, support longer sport participation, and reduce injury and burnout risks associated with early specialisation (11, 12). These concepts are reflected in Local TID (L-TID) programmes led by local governments in Japan, which originated in Fukuoka Prefecture and later expanded nationwide (13–15). A key feature of the L-TID model is early multi-sport exposure and “sport matching”, where individuals are aligned with sports based on their aptitude, facilitating sport transitioning to more suitable pathways after initial evaluation (14, 16, 17). This emphasis on early-stage matching during key growth periods differs from many Western approaches, which often focus on talent transfer at later stages. However, because the pubertal period is highly dynamic, effective implementation requires careful consideration of biological maturation, yet longitudinal evidence linking maturity and physical development within Japanese L-TID cohorts remains limited.

To date, many longitudinal studies have used the growth spurt, specifically peak height velocity (PHV), as a benchmark for examining physical development during maturation (18, 19). Recent meta-analyses have established robust reference values for age at PHV in male and female athletes (20, 21), providing a standardised framework for monitoring growth. Due to the non-linear nature of physical development, established models such as the Youth Physical Development (YPD) have become central to structuring long-term physical development (22). The YPD model emphasises that the timing of PHV marks a critical transition from predominantly neural-driven development to muscle strength and power improvements mediated by hormonal changes (22, 23). Navigating this transition presents unique challenges for youth athletes. Rapid anthropometric changes around PHV may temporarily inhibit motor control, a phenomenon known as “adolescent awkwardness” (24). In addition, pain and injury risk may increase when skeletal growth outpaces the adaptation of muscles and tendons (25, 26). Therefore, understanding the specific physiological and motor control shifts that occur relative to PHV is critical for practitioners working with youth populations.

In Japan, previous researchers have examined maturity-related differences in motor coordination within single sports such as soccer (27, 28) and sprinting (29). However, multidimensional longitudinal evidence including multiple fitness components remains insufficient. Moreover, most findings are derived from Caucasian athletes in elite academy contexts. Given potential ethnic variations in growth tempo and body composition between Asian and Caucasian populations (20, 21), it may be inappropriate to extrapolate Western benchmarks to Japanese youth. However, longitudinal data tracking the physical fitness of Asian (including Japanese) youth athletes during puberty are comparatively scarce (20, 21). Consequently, there is a clear need for longitudinal evidence specific to this population to determine whether disruptions in motor control related to maturity occur in a consistent or maturity-specific manner among Japanese youth. By analysing a unique 20-year dataset from the Fukuoka L-TID programme, this study addresses the regional knowledge gap in Asia by directly examining the influence of biological maturation and PHV timing on both absolute performance gains and the longitudinal stability of individual rankings. Specifically, the investigation tests whether the performance volatility associated with adolescent awkwardness follows a pattern specific to maturity across Early, Average, and Late groups; this is an empirical question previously unexplored using multidimensional longitudinal data in this population.

To the best of our knowledge, this is the first study to draw on 20 years of multidimensional data from a government-supported TID programme to examine the longitudinal development of anthropometry and physical fitness in talented Japanese youth athletes across puberty. Developmental patterns are characterised across multiple fitness components, which are categorised by both sex and biological maturity status. By analysing longitudinal data from a Fukuoka L-TID cohort with superior athletic ability, this study provides evidence to inform early-stage TID programmes and supports the design of more effective, maturity-informed athlete development systems in Japan and internationally.

## 2 Materials and methods

### 2.1 Subjects

This study was approved by the Institutional Ethics Committee of the first author (Approval Number: 2022-012) and was conducted in accordance with the Declaration of Helsinki. A total of 383 participants (182 males, 201 females) graduated from the Fukuoka L-TID programme between 2005 and 2019 (13, 14). From this cohort, a final sample of 96 participants (43 males, 53 females) was selected based on the availability of continuous, longitudinal records for height, body mass, and physical fitness tests from the Grade 4 (G4) to Grade 9 (G9).

The high attrition rate (approximately 75%) was primarily due to our strict inclusion criteria; only athletes with a complete 6-year data record were included to ensure the integrity of the longitudinal analysis. Furthermore, as the programme follows a multi-sport concept (13, 14), participants were permitted to prioritise important competitions or practices in their primary sports over the annual tests. While this flexibility supported athlete development, it resulted in missing data points for some individuals, leading to their exclusion from the final dataset.

Under this model, all participants followed a standard curriculum that included systematic experience in about 10 different sports (e.g., athletics, wrestling, and fencing). This “sport-sampling” and “sport-matching” approach continued until the end of G9, when participants chose a specific sport based on their results (13, 14). This design ensured that the development patterns observed in this study were not affected by early sport specialisation.

### 2.2 The timing of PHV and maturity-based categorisation

Somatic maturity was evaluated using the timing of peak height velocity (PHV) as a surrogate indicator (18). Annual growth rates were calculated from longitudinal height data from G4 to G9. For each participant, the “PHV period” was defined as the 12-month interval corresponding to the maximum absolute increase in height (cm/year). Although the use of annual height measurements is a standard longitudinal approach, it is important to acknowledge that this interval may introduce potential misclassification of the PHV timing in individuals with particularly rapid or brief growth spurts. This inherent limitation of measurement frequency was considered during the interpretation of maturity-based categorisations.

Participants were categorised into three maturity groups as Early, Average, or Late based on the timing of PHV relative to Japanese population norms (30, 31) (Table 1). While the Average maturity group was defined to balance sample sizes, some imbalances remained due to the inherent distribution of the cohort.

**Table 1:** Maturity categorisation by maturity status based on PHV timing.

Sex	Maturity Group	School grade level				
		G4-G5	G5-G6	G6-G7	G7-G8	G8-G9
Male	Early (*n* = 16)	3	13			
	Average (*n* = 13)			13†		
	Late (*n* = 14)				12	2
Female	Early (*n* = 22)	22				
	Average (*n* = 24)		24†			
	Late (*n* = 7)			4	2	1

G, school grade. The school grade levels marked with a dagger (†) represent the Average maturity group based on Japanese population norms. The periods before and after these levels are defined as the Early and Late maturity groups, respectively.

### 2.3 Anthropometric and physical fitness assessments

The testing included height, body mass, 25-m sprint, standing broad jump (SBJ), reaction time (RT), and 20-m shuttle run (SR). Height, body mass, SBJ, and SR were administered according to the national guidelines (32). To ensure high data quality, height and body mass were measured to the nearest 0.1 cm and 0.1 kg, respectively, using calibrated stadiometers and scales in accordance with these standardised protocols. Specifically, the SBJ assessed explosive lower-limb power via horizontal displacement from a stationary start, while SR evaluated endurance through maximal incremental running involving repeated acceleration and deceleration. Notably, SR was not administered at G4 in accordance with the national guidelines (32). At this developmental stage (approximately 9–10 years old), children typically lack the neurocognitive maturation required for precise pacing strategies and the rhythmic synchronisation necessary for incremental exercise. Remaining variables followed validated protocols (33–35). Within these protocols, the 25-m sprint measured maximal sprint speed, while RT assessed neurocognitive processing speed and the initiation of a motor response, categorised into response initiation and motor execution phases (35, 36). To minimise potential human error associated with manual timing, both the 25-m sprint and RT were measured using automated mechanical timing systems. All testing sessions were supervised by trained physical education teachers and programme staff to ensure standardisation. Annual changes between consecutive grades in each variable were calculated and expressed as Δ.

### 2.4 Statistical analyses

Data in tables are presented as *mean* ± *SD* to describe the distribution, while data in figures are presented as means with 95% confidence intervals (*CI*) to facilitate the comparison of differences. Data normality was assessed using the *Shapiro–Wilk test*. While some variables exhibited deviations from normality, a two-way repeated measures *ANOVA* was employed as it is generally robust to such violations in longitudinal studies of this scale (*n* = 96). The model examined the effects of maturity group (Early, Average, Late; between-subjects) and grade (G4-G9; within-subjects) on the variables. Mauchly's test assessed the assumption of sphericity, with the *Greenhouse-Geisser correction* applied when violations occurred. The primary focus was the Group × Grade interaction to determine if development patterns differed by maturity timing. Significant main and interaction effects were analysed using *Bonferroni post-hoc tests*. Additionally, considering the limited sample size in certain sub-groups (e.g., female Late maturation group, *n* = 7), non-parametric tests were performed to ensure the stability and validity of the results. The *Friedman test* was used to evaluate longitudinal changes within each maturity group, followed by *Bonferroni-corrected pairwise comparisons*. The *Kruskal–Wallis test* was applied to compare physical fitness variables among the three maturity groups at each grade level.

Effect sizes were expressed as partial eta squared (*ηp²*) and interpreted as small (0.01), medium (0.06), or large (0.14) (37). Given the relatively small sample sizes in certain subgroups (e.g., the female Late maturity group), *ηp²* was reported alongside *p*-values to evaluate practical significance and ensure that meaningful effects were not overlooked due to limited statistical power. Consequently, findings specific to the female Late maturity group (*n* = 7) should be interpreted with particular caution. The Bonferroni correction was applied to all *post-hoc* comparisons, while conservative and appropriate for controlling familywise error rate, cannot fully compensate for the increased risk of Type II error and unstable effect size estimates associated with this subgroup size. Although ICCs were not calculated for this retrospective dataset, reliability was ensured through standardised national protocols and objective mechanical timing systems.

To evaluate the ranking stability across groups of varying sizes and under conditions of potential non-normal distribution, both Pearson's *r* and Kendall's *τ* were calculated between initial (G4; G5 for SR) and final (G9) measurements to assess the relationship and predictive ability of each variable. In particular, Pearson's *r* quantified the linear association between initial and final performance, while Kendall's *τ* assessed the stability of individual performance rankings for each physical fitness variable within each maturity group over the study period. The coefficient of determination (*r^2^*) was used to indicate the proportion of variance explained. Correlation magnitudes were interpreted as moderate (0.40–0.69), strong (0.70–0.89), or very strong (0.90–1.00) (38). Statistical significance was set at *p* < 0.05, and all analyses were performed using IBM SPSS Statistics (version 29.0).

## 3 Results

### 3.1 Anthropometric development

**Figure 1:**
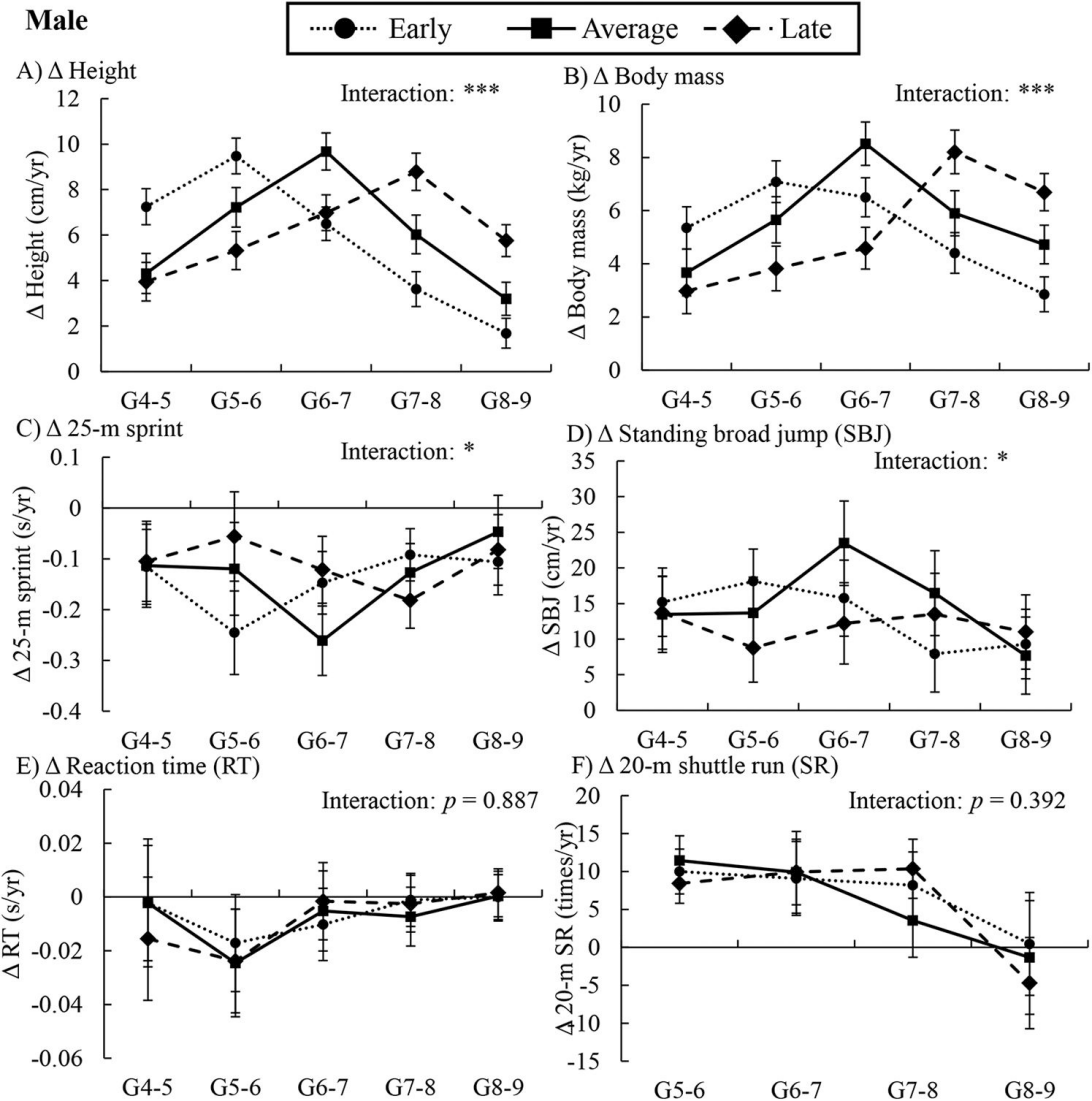
Annual changes in anthropometric and physical fitness variables by maturity groups (early, average, or late) in male participants. **(A)** Height, **(B)** Body mass, **(C)** 25-m sprint, **(D)** Standing broad jump (SBJ), **(E)** Reaction time (RT), and **(F)** 20-m shuttle run (SR). For 20-m SR, sample sizes are smaller because the test began in Grade 5 and followed the programme's flexible multi-sport participation policy: Early (*n* = 11), Average (*n* = 9), and Late (*n* = 14). To ensure statistical robustness, findings for variables with limited sample sizes were further validated using Friedman and Kruskal–Wallis tests. Data are presented as mean ± 95% confidence intervals (*CI*). The *p*-values represent the Group × Grade interaction from the two-way repeated-measures analysis of variance (*ANOVA*). *p < 0.05, ***p < 0.001.

**Figure 2:**
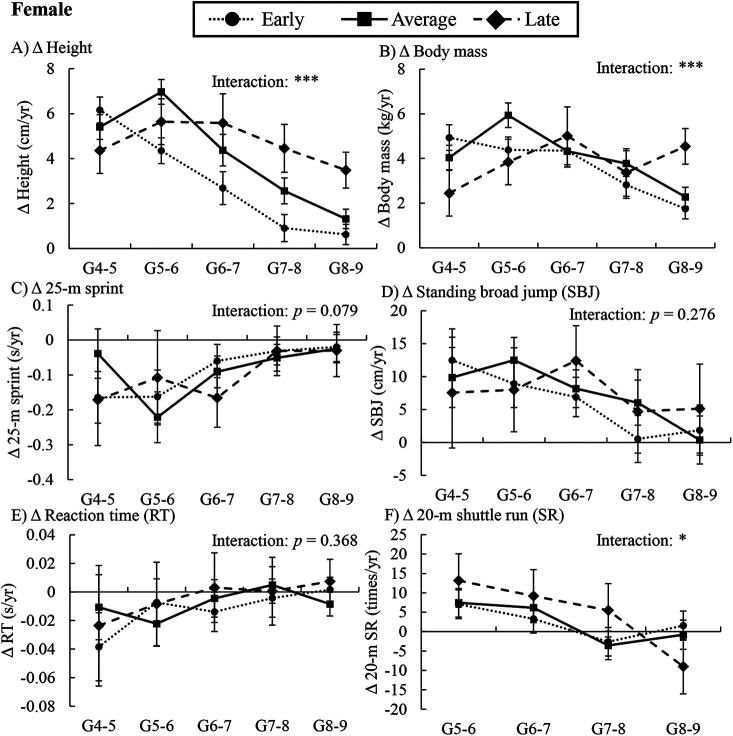
Annual changes in anthropometric and physical fitness variables by maturity groups (early, average, or late) in female participants. **(A)** Height, **(B)** Body mass, **(C)** 25-m sprint, **(D)** Standing broad jump (SBJ), **(E)** Reaction time (RT), and **(F)** 20-m shuttle run (SR). For 20-m SR, sample sizes are smaller because the test began in Grade 5 and followed the programme's flexible multi-sport participation policy: Early (*n* = 21), Average (*n* = 21), and Late (*n* = 6). To ensure statistical robustness, findings for variables with limited sample sizes were further validated using Friedman and Kruskal–Wallis tests. Data are presented as mean ± 95% confidence intervals (*CI*). The *p*-values represent the Group × Grade interaction from the two-way repeated-measures analysis of variance (*ANOVA*). *p < 0.05, ***p < 0.001.

Tables 2, 3 show the annual changes across all variables, and Figures 1, 2 illustrate sex-specific developmental patterns. For both sexes, highly significant Group × Grade interactions were observed for annual increments in height and body mass (Δ Height, Δ Body mass; *p* < 0.001). These interactions were supported by large effect sizes for Δ Height (males: *ηp²* = 0.644; females: *ηp²* = 0.353) and Δ Body mass (males: *ηp²* = 0.279; females: *ηp²* = 0.132). These substantial effect sizes verify that the longitudinal growth trajectories were successfully captured by the maturity-based categorisations, despite the stratified sample sizes. In males, the Early maturity group reached PHV during G5-G6 (9.5 ± 2.0 cm/year), while the Average and Late maturity groups peaked at G6-G7 (9.7 ± 1.3 cm/year) and G7-G8 (8.8 ± 1.1 cm/year), respectively (Figure 1A). Similarly, in females, the Early maturity group reached PHV at G4-G5 (6.2 ± 1.7 cm/year), the Average maturity group at G5-G6 (7.0 ± 1.1 cm/year), and the Late maturity group exhibited a prolonged peak spanning G5-G6 (5.6 ± 0.8 cm/year) and G6-G7 (5.6 ± 2.8 cm/year) (Figure 2A). These results confirm that longitudinal growth patterns aligned with the predefined maturity categorisations for both sexes.

**Table 2:** Annual changes in anthropometric and physical fitness variables by maturity groups (early, average, or late) in male participants.

Variable (Unit)	Maturity group	School grade level															*ANOVA* results					
		G4-G5			G5-G6			G6-G7			G7-G8			G8-G9			Interaction		Main Effect		Main Effect	
		*Mean*		*SD*	*Mean*		*SD*	*Mean*		*SD*	*Mean*		*SD*	*Mean*		*SD*	(Group × Grade)		(Grade)		(Group)	
Δ Height (cm/year)	Early (*n* = 16)	7.3	±	2.1	**9**.**5**	±	2.0	6.5	±	1.9	3.6	±	1.7	1.7	±	0.9	*F* = 36.25		*F* = 56.86		*F* = 1.89	
	Average (*n* = 13)	4.3	±	1.5	7.2	±	1.3	**9**.**7**	±	1.3	6.0	±	1.7	3.2	±	1.3	*p* < 0.001	***	*p* < 0.001	***	*p* = 0.164	
	Late (*n* = 14)	4.0	±	0.7	5.3	±	1.1	7.0	±	1.0	**8**.**8**	±	1.1	5.8	±	1.7	*η_p_²* = 0.644	††	*η_p_²* = 0.587	††	*η_p_²* = 0.086	†
Δ Body mass (kg/yr)	Early (*n* = 16)	5.4	±	1.5	**7**.**1**	±	3.8	6.5	±	4.7	4.4	±	1.7	2.9	±	2.5	*F* = 7.76		*F* = 6.46		*F* = 1.51	
	Average (*n* = 13)	3.7	±	1.6	5.7	±	1.9	**8**.**5**	±	2.4	5.9	±	2.9	4.7	±	3.1	*p* < 0.001	***	*p* = 0.001	***	*p* = 0.233	
	Late (*n* = 14)	3.0	±	1.0	3.8	±	1.3	4.6	±	1.4	**8**.**2**	±	2.1	6.7	±	1.9	*η_p_²* = 0.279	††	*η_p_²* = 0.139	††	*η_p_²* = 0.070	†
Δ 25-m sprint (s/yr)	Early (*n* = 16)	−0.12	±	0.19	**−0**.**25**	±	0.19	−0.15	±	0.11	−0.09	±	0.10	−0.11	±	0.11	*F* = 2.80		*F* = 2.66		*F* = 4.24	
	Average (*n* = 13)	−0.11	±	0.11	−0.12	±	0.14	**−0**.**26**	±	0.15	−0.13	±	0.12	−0.05	±	0.12	*p* = 0.012	*	*p* = 0.048	*	*p* = 0.021	*
	Late (*n* = 14)	−0.10	±	0.12	−0.06	±	0.15	−0.12	±	0.10	**−0**.**18**	±	0.08	−0.08	±	0.15	*η_p_²* = 0.123	†	*η_p_²* = 0.062	†	*η_p_²* = 0.175	††
Δ SBJ (cm/year)	Early (*n* = 16)	15.2	±	7.2	**18**.**1**	±	10.7	15.8	±	10.0	7.9	±	10.2	9.3	±	7.3	*F* = 2.14		*F* = 3.11		*F* = 3.09	
	Average (*n* = 13)	13.5	±	14.2	13.7	±	7.5	**23**.**5**	±	10.7	16.5	±	9.8	7.7	±	9.3	*p* = 0.035	*	*p* = 0.017	*	*p* = 0.057	
	Late (*n* = 14)	**13**.**7**	±	5.7	8.8	±	7.9	12.2	±	11.1	13.5	±	11.7	11.0	±	12.0	*η_p_²* = 0.097	†	*η_p_²* = 0.072	†	*η_p_²* = 0.134	†
Δ RT (s/yr)	Early (*n* = 16)	−0.002	±	0.040	**−0**.**017**	±	0.039	−0.010	±	0.028	−0.001	±	0.022	0.000	±	0.015	*F* = 0.34		*F* = 2.95		*F* = 0.43	
	Average (*n* = 13)	−0.002	±	0.035	**−0**.**025**	±	0.028	−0.005	±	0.027	−0.007	±	0.012	0.000	±	0.018	*p* = 0.887		*p* = 0.046	*	*p* = 0.654	
	Late (*n* = 14)	−0.016	±	0.051	**−0**.**024**	±	0.039	−0.002	±	0.025	−0.003	±	0.023	0.002	±	0.017	*η_p_²* = 0.017		*η_p_²* = 0.069	†	*η_p_²* = 0.021	
Δ SR (times/yr)	Early (*n* = 11)	n.a.			**10.0**	±	5.0	9.1	±	3.6	8.2	±	6.3	0.5	±	9.3	*F* = 1.05		*F* = 14.01		*F* = 0.34	
	Average (*n* = 9)	n.a.			**11.4**	±	1.9	9.9	±	10.8	3.6	±	7.7	−1.3	±	7.0	*p* = 0.392		*p* < 0.001	***	*p* = 0.713	
	Late (*n* = 14)	n.a.			8.4	±	5.8	9.9	±	8.3	**10** **.** **4**	±	7.4	−4.7	±	13.9	*η_p_²* = 0.063	†	*η_p_²* = 0.311	††	*η_p_²* = 0.022	

Values are expressed as *Mean* ± *SD*. Bold values indicate the peak annual changes (maximal velocity or improvement) within each maturity group. SBJ, standing broad jump; RT, reaction time; SR, 20-m shuttle run; n.a., not available (SR testing commenced in G5). For 25-m sprint and RT, negative values denote performance improvement (time reduction); for SR, negative values denote a decline in performance. The *Greenhouse-Geisser* adjustment was applied to *F*-tests for variables where the assumption of sphericity was violated. *p < 0.05, ***p < 0.001, Effect sizes (*ηp²*) are qualitatively described as medium († ≥ 0.06) or large (†† ≥ 0.14).

**Table 3:** Annual changes in anthropometric and physical fitness variables by maturity group (early, average, or late) in female participants.

Variable	Maturity group	School grade level															*ANOVA* results					
(Unit)		G4-G5			G5-G6			G6-G7			G7-G8			G8-G9			Interaction		Main Effect		Main Effect	
		*Mean*		*SD*	*Mean*		*SD*	*Mean*		*SD*	*Mean*		*SD*	*Mean*		*SD*	(Group × Grade)		(Grade)		(Group)	
Δ Height (cm/year)	Early (*n* = 22)	**6**.**2**	±	1.7	4.4	±	1.6	2.7	±	1.2	0.9	±	0.7	0.6	±	0.6	*F* = 13.62		*F* = 76.83		*F* = 15.13	
	Average (*n* = 24)	5.4	±	1.0	**7**.**0**	±	1.1	4.4	±	1.8	2.6	±	1.5	1.3	±	1.0	*p* < 0.001	***	*p* < 0.001	***	*p* < 0.001	***
	Late (*n* = 7)	4.4	±	1.1	**5**.**6**	±	0.8	**5**.**6**	±	2.8	4.5	±	2.5	3.5	±	2.0	*η_p_²* = 0.353	††	*η_p_²* = 0.606	††	*η_p_²* = 0.377	††
Δ Body mass (kg/yr)	Early (*n* = 22)	**4**.**9**	±	1.7	4.4	±	2.5	4.3	±	2.0	2.8	±	1.6	1.7	±	1.7	*F* = 3.80		*F* = 6.42		*F* = 1.78	
	Average (*n* = 24)	4.0	±	1.4	**5**.**9**	±	2.2	4.3	±	2.4	3.8	±	1.9	2.3	±	1.8	*p* < 0.001	***	*p* < 0.001	***	*p* = 0.179	
	Late (*n* = 7)	2.4	±	1.2	3.8	±	1.4	**5**.**0**	±	2.9	3.4	±	0.8	4.5	±	2.0	*η_p_²* = 0.132	††	*η_p_²* = 0.114	†	*η_p_²* = 0.066	†
Δ 25-m sprint (s/yr)	Early (*n* = 22)	**−0**.**164**	±	0.151	−0.162	±	0.174	−0.060	±	0.093	−0.031	±	0.091	−0.020	±	0.108	*F* = 2.08		*F* = 6.34		*F* = 0.56	
	Average (*n* = 24)	−0.039	±	0.176	**−0**.**221**	±	0.186	−0.091	±	0.125	−0.051	±	0.105	−0.025	±	0.088	*p* = 0.079		*p* = 0.002	**	*p* = 0.573	
	Late (*n* = 7)	**−0**.**171**	±	0.222	−0.108	±	0.159	−0.165	±	0.115	−0.031	±	0.038	−0.030	±	0.099	*η_p_²* = 0.077	†	*η_p_²* = 0.113	†	*η_p_²* = 0.022	
Δ SBJ (cm/year)	Early (*n* = 22)	**12**.**5**	±	11.3	8.9	±	8.1	6.9	±	7.3	0.5	±	7.5	1.9	±	8.0	*F* = 1.26		*F* = 5.81		*F* = 1.43	
	Average (*n* = 24)	9.9	±	10.3	**12**.**5**	±	9.2	8.2	±	6.4	6.0	±	8.7	0.4	±	9.9	*p* = 0.276		*p* < 0.001	***	*p* = 0.249	
	Late (*n* = 7)	7.6	±	12.9	8.0	±	5.9	**12**.**4**	±	7.8	4.7	±	9.7	5.1	±	8.0	*η_p_²* = 0.048		*η_p_²* = 0.104	†	*η_p_²* = 0.054	
Δ RT (s/yr)	Early (*n* = 22)	**−0**.**038**	±	0.056	−0.007	±	0.041	−0.014	±	0.031	−0.004	±	0.023	0.002	±	0.021	*F* = 1.09		*F* = 2.58		*F* = 2.35	
	Average (*n* = 24)	−0.011	±	0.042	**−0**.**022**	±	0.038	−0.004	±	0.032	0.005	±	0.038	−0.009	±	0.020	*p* = 0.368		*p* = 0.069		*p* = 0.106	
	Late (*n* = 7)	**−0**.**024**	±	0.088	−0.008	±	0.029	0.003	±	0.035	0.001	±	0.029	0.007	±	0.017	*η_p_²* = 0.042		*η_p_²* = 0.049		*η_p_²* = 0.086	
Δ SR (times/yr)	Early (*n* = 21)	n.a.			**7** **.** **0**	±	8.9	3.3	±	8.3	−2.6	±	8.2	1.5	±	10.1	*F* = 2.29		*F* = 12.46		*F* = 2.17	
	Average (*n* = 21)	n.a.			**7** **.** **4**	±	7.1	6.1	±	7.9	−3.6	±	9.2	−0.8	±	7.5	*p* = 0.039	*	*p* < 0.001	***	*p* = 0.126	
	Late (*n* = 6)	n.a.			**13.2**	±	10.9	9.2	±	9.9	5.5	±	4.4	−9.0	±	5.4	*η_p_²* = 0.092	†	*η_p_²* = 0.217	††	*η_p_²* = 0.088	†

Values are expressed as *Mean* ± *SD*. Bold values indicate the peak annual changes (maximal velocity or improvement) within each maturity group. SBJ, standing broad jump; RT, reaction time; SR, 20-m shuttle run; n.a., not available (SR testing commenced in G5). For 25-m sprint and RT, negative values denote performance improvement (time reduction); for SR, negative values denote a decline in performance. The *Greenhouse-Geisser* adjustment was applied to *F*-tests for variables where the assumption of sphericity was violated. *p < 0.05, ***p < 0.001, Effect sizes (*ηp²*) are qualitatively described as medium († ≥ 0.06) or large (†† ≥ 0.14).

### 3.2 Physical fitness trajectories by sex

In males, significant Group × Grade interactions were found for the 25-m sprint (*p* = 0.012, *ηp²* = 0.123) and the SBJ (*p* = 0.035, *ηp²* = 0.097). As shown in Figures 1C,D, the magnitude of performance improvements in sprint speed and explosive power metrics was synchronised with the timing of PHV for each maturity group. The medium-to-large effect sizes indicate that biological maturation significantly influenced the developmental trajectories of these power-related components. In contrast, no significant interactions were observed for RT or SR in males (Figures 1E,F). In females, a significant interaction was found only for SR (*p* = 0.039, *ηp²* = 0.092), where the Late maturity group showed greater improvement during G5-G6 (13.2 ± 10.9 times/year). However, SR subsequently declined significantly across all female maturity groups by G8-G9 (Figure 2F). Despite the smaller sample size in the female Late group (*n* = 7), the observed medium effect size suggests a meaningful developmental constraint during this period. No significant interactions were observed for the 25-m sprint, SBJ, or RT (*p* > 0.05, *ηp²* = 0.042–0.077), indicating more uniform developmental patterns across maturity groups for these variables in females (Figures 2C–E).

Non-parametric analysis further supported these findings. For the female late-maturation group, the *Friedman test* indicated a significant chronological effect on SR performance changes [*χ^2^*(3) = 11.000, *p* = 0.012]. *Post-hoc pairwise comparisons* revealed that the magnitude of performance change in the G8–G9 period was significantly different from those in the G5–G6 (*p* = 0.022) and G7–G8 (*p* = 0.044) periods. Furthermore, the *Kruskal–Wallis test* confirmed significant inter-group differences in SR during the G8–G9 interval (*p* = 0.031).

### 3.3 Stability of individual performance rankings

Table 4 presents both Pearson's *r* and Kendall's *τ* to distinguish between the maintenance of absolute performance levels and the stability of individual rankings within maturity groups. The findings revealed a moderate-to-strong correlation between height, body mass, and SR across all groups (*r* = 0.41–0.89, *τ* = 0.27–0.77), with the highest stability observed in the Average maturity group in males (*r* = 0.84, *τ* = 0.75, *r^2^* = 69.7%). In contrast, the remaining physical fitness variables showed a notable divergence between absolute performance progression and ranking stability. In the case of 25-m sprint, both *r* and *τ* were consistently low in the Early and Late maturity groups (*r* ≤ 0.17, *τ* ≤ 0.12, *p* > 0.05). Similarly, for the SBJ, the Early maturity group and male Average group showed low ranking stability (*r* ≤ 0.29, *τ* ≤ 0.22, *p* > 0.05), whereas significant stability was maintained in the female Average group (*r* = 0.52, *τ* = 0.41, *p* < 0.01) and the male Late maturity group (*τ* = 0.42, *p* < 0.05). For RT, ranking stability was markedly low in the Average maturity group in both sexes (*r* ≤ 0.22, *τ* ≤ 0.11, *p* > 0.05). Although the Early and Late groups showed moderate-to-strong Pearson correlations (*r* = 0.58–0.70), the correspondingly lower Kendall's *τ* values (e.g., female Late: *τ* = 0.14) indicate that individual relative standing undergoes substantial re-ranking during the peak growth phase, even where absolute performance shows some degree of consistency.

**Table 4:** Pearson and Kendall correlation coefficients of anthropometric and physical fitness variables between entry (grade 4/5) and exit (grade 9) stratified by maturity groups and gender.

Variable	Correlation coefficient	Early						Average						Late					
		Males (*n* = 16)			Females (*n* = 22)			Males (*n* = 13)			Females (*n* = 24)			Males (*n* = 14)			Females (*n* = 7)		
		[PHV@G4-G6]			[PHV@G4-G5]			[PHV@G6-G7]			[PHV@G5-G6]			[PHV@G7-G9]			[PHV@G6-G9]		
		*r/τ*	*p*	*r^2^ (%)*	*r/τ*	*p*	*r^2^ (%)*	*r/τ*	*p*	*r^2^ (%)*	*r/τ*	*p*	*r^2^ (%)*	*r/τ*	*p*	*r^2^ (%)*	*r/τ*	*p*	*r^2^ (%)*
Height (G4 vs. G9)	Pearson's *r*	0.63	**	40.2	0.65	**	42.1	0.86	**	74.0	0.60	**	36.1	0.79	**	62.7	0.64		41.3
	Kendall's *τ*	0.45	*	-	0.43	**	-	0.72	**	-	0.37	*	-	0.62	**	-	0.43		-
Body mass (G4 vs. G9)	Pearson's *r*	0.55	*	30.4	0.59	**	35	0.76	**	58.2	0.50	*	25.0	0.89	**	79.4	0.41		16.6
	Kendall's *τ*	0.39	*	-	0.27		-	0.64	**	-	0.34	*	-	0.77	**	-	0.39		-
25-m sprint (G4 vs. G9)	Pearson's *r*	0.17		2.8	0.03		0.1	0.48		23.3	0.57	**	32.1	0.14		2.0	0.04		0.2
	Kendall's *τ*	0.08		-	0.07		-	0.43	*	-	0.31	*	-	0.12		-	−0.14		-
SBJ (G4 vs. G9)	Pearson's *r*	−0.08		0.6	0.04		0.2	0.29		8.3	0.52	**	27.5	0.51		25.6	0.46		21.0
	Kendall's *τ*	−0.19		-	−0.01		-	0.22		-	0.41	**	-	0.42	*	-	0.29		-
RT (G4 vs. G9)	Pearson's *r*	0.63	**	39.7	0.58	**	33.5	0.09		0.8	0.22		4.9	0.70	**	49.4	0.58		33.5
	Kendall's *τ*	0.43	*	-	0.47	**	-	0.01		-	0.11		-	0.33		-	0.14		-
SR (G5 vs. G9) †	Pearson's *r*	0.71	*	51.0	0.66	**	43.4	0.84	**	69.7	0.77	**	59.6	0.78	**	61.3	0.66		43.7
	Kendall's *τ*	0.34		-	0.51	**	-	0.75	**	-	0.52	**	-	0.59	**	-	0.69		-

PHV, peak height velocity; G, school grade; *r*, correlation coefficient (Pearson's); *τ*, correlation coefficient (Kendall's); *r^2^*, coefficient of determination; SBJ, standing broad jump; RT, reaction time; SR, 20-m shuttle run. Maturity groups (Early/Average/Late) are based on the grade of PHV. SR was analysed between G5 and G9. † Due to the later introduction of SR testing and the programme's multi-sport flexibility, sample sizes for SR were smaller than other variables: *n* = 11, 9, 14 for Early, Average, and Late males, and *n* = 21, 21, 6 for Early, Average, and Late females, respectively.

* *p* < 0.05.

** *p* < 0.01.

## 4 Discussion

To our knowledge, this study is the first to leverage 20 years of multidimensional longitudinal data from a government-supported Japanese L-TID programme to examine physical fitness development relative to biological maturation in talented Japanese youth athletes. The primary finding was that improvements in sprint speed (25-m sprint) and explosive power (SBJ) among male participants were closely synchronised with the timing of PHV, a pattern supported by medium-to-large interaction effects across anthropometric and physical fitness variables (Table 2; Figure 1). In contrast, endurance performance (SR) among female cohorts tended to decline following PHV, a pattern supported by a medium interaction effect suggesting that biological maturation may act as a transient physiological constraint for females (9, 31) (Table 3; Figure 2). Importantly, the rate of RT improvement remained independent of maturity-related changes in both sexes, as evidenced by a large main effect of Grade (Tables 2, 3). This pattern points towards steady, age-related neurocognitive development that reaches a plateau by the final observation period between G8 and G9, where annual changes approached zero across all groups. However, correlation analyses demonstrated that maturity timing influenced the stability of individual performance rankings between programme entry (G4; G5 for SR) and programme exit (G9) (Table 4). Specifically, the Average maturity group showed lower ranking stability because many participants were assessed during a period of pronounced neural and physical transition around PHV. For the female Average maturity group, the adaptation process may be prolonged because they must manage both neuromuscular re-integration and the physical constraint of increased body mass. Consequently, these findings suggest that biological maturation can make prediction of later performance from early measurements particularly challenging when assessments occur close to PHV.

### 4.1 Validity of maturity assessment based on PHV

In adolescent athletes, biological age often differs significantly from chronological age (9). Thus, maturity assessment is essential for identifying long-term athletic potential rather than just evaluating current talent (5). In this study, we used the timing of PHV as a surrogate indicator for biological maturation. By categorising participants into Early, Average, and Late maturity groups, we observed that peak height growth occurred in G6–G7 for males and G5–G6 for females which broadly aligns with longitudinal standards for the Japanese population (30). Although the peak magnitudes observed (9.7 ± 1.3 cm/year for males; 7.0 ± 1.1 cm/year for females) were slightly lower than reference values, this is likely attributable to the annual measurement interval, which can underestimate instantaneous peak velocity.

Overall, these results suggest that PHV-based categorisation is a feasible approach for monitoring non-linear development in Japanese L-TID cohorts. Although using general population norms (30, 31) is a limitation, it was necessary as athlete-specific standards for Japanese youth do not yet exist. This study serves as a foundational step toward establishing such benchmarks. However, identifying PHV based on annual changes is primarily retrospective (9). To improve practical utility, future initiatives should consider estimating height velocity from more frequent measurements (e.g., quarterly), which may allow prospective identification of the PHV period in real time. Alternatively, skeletal age assessment using x-ray-based methods (e.g., Tanner-Whitehouse) provides a maturity estimate at the specific time of measurement (9, 39). Although skeletal age is often considered the “gold standard” method (40), its application in youth sport is limited by radiation exposure, the need for specialised facilities, and cost. Therefore, for large-scale TID programmes, more frequent height monitoring may be the most feasible strategy.

Crucially, increasing measurement frequency (e.g., monthly vs. quarterly) may enable earlier detection of the growth-spurt onset than retrospective annual analysis. However, empirical studies are needed to verify the predictive accuracy and specific effectiveness of such high-frequency monitoring for identifying growth-spurt onset in athletic populations.

### 4.2 Sex-specific development patterns

This study revealed clear sex differences in how biological maturation relates to physical performance. In males, peak improvements in sprint speed and explosive power were closely synchronised with the PHV period (Figures 1C,D). This pattern provides empirical support for the YPD model, which suggests that pubertal hormonal changes (specifically increases in testosterone) combined with somatic growth act as physiological accelerators of strength, power, and speed (22, 23). These researchers emphasize that increases in muscle-tendon stiffness and muscle mass during male puberty may interact synergistically with neural development, leading to rapid gains in explosive tasks.

In contrast, females did not show a similar maturity-linked boost in sprint speed or explosive power (Table 3). However, in alignment with YPD considerations for female athletes, this does not necessarily indicate limited power development. It is plausible that absolute power capacity increases, but significant gains in body mass and fat mass reduce relative performance in weight-bearing tasks, thereby masking improvements. The significant interaction in SR (*p* = 0.039), where performance declined after PHV (Figure 2F), strongly supports this interpretation. Since SR requires repeated accelerations and decelerations, increased body mass and fat mass may elevate the metabolic cost of locomotion and act as a transient physiological constraint (9, 31).

Furthermore, adolescent awkwardness, which is a temporary disruption in motor control during rapid growth, may further influence these patterns (24, 41). Although this phase can affect both sexes, females may face a more complex transition during the post-PHV phase. In particular, females may need to adapt concurrently to neuromuscular re-integration and to a body that is mechanically and metabolically more demanding to move. Thus, the observed decline in endurance performance likely reflects a transient period of physiological and neuromuscular adaptation rather than a loss of long-term athletic potential.

### 4.3 Stability of neurocognitive development

In contrast to power and endurance, RT exhibited no significant Group × Grade interaction for either sex (males: *p* = 0.887; females: *p* = 0.368) and improved independently of maturation status (Tables 2, 3). This suggests that neurocognitive development follows a stable timeline that remains relatively independent of the rapid structural changes associated with puberty. This aligns with the YPD model indicating that many neural functions reach adult-like levels earlier than peak somatic growth (22, 23). Notably, while performance generally improved throughout the study, the rate of improvement slowed toward the end of junior-high school, with several groups reaching a plateau between G8 and G9 where annual changes approached zero.

However, a discrepancy exists between this quantitative stability and the individual ranking trajectories. As shown in Table 4, the discrepancy between Pearson's *r* and Kendall's *τ* in RT results warrants careful interpretation. Although the Average maturity group improved at a comparable rate to other groups, individual RT rankings were less stable during the G4-G9 period. Even in the Early and Late maturity groups, which showed moderate-to-strong Pearson correlations (*r* = 0.58–0.70), the correspondingly lower Kendall's *τ* values (e.g., female Late: *τ* = 0.14) reveal that intra-group ranking order remained highly volatile.

This may reflect the timing of PHV relative to the final measurement. The Early maturity group likely had more time to stabilise motor control after their growth spurt, whereas the Average maturity group reached PHV during the programme, leaving less time for neuromuscular re-integration before the final measurement at G9. This implies that although neurocognitive processing may improve with age, the individual trajectory of development and the timing of neuromuscular re-integration vary substantially among talented athletes. Consequently, individual performance in the Average maturity group may have been more susceptible to temporary fluctuations associated with adolescent awkwardness (24, 41), leading to weaker correlations. In contrast, the Late maturity group's rankings may have remained more stable because many had not yet entered the most volatile phase of motor disruption by G9.

Recent research also supports the idea that the pre-pubertal period constitutes a “critical window of neuroplasticity” during which the central nervous system is most receptive to motor skill acquisition and neuromuscular control (42). Since RT showed substantial improvements during the earlier grades before the progress began to level off toward G9 (Figures 1E, 2E), it may represent a stable attribute that is less influenced by maturity-related fluctuations. From a TID perspective, such neurocognitive indicators may be valuable as they allow for the independent assessment of neurotransmission and muscle contraction processes (36) and are inherently difficult to improve significantly once the primary neural developmental phase concludes (22, 23). Thus, identifying individuals with superior reaction speed and movement intelligence during early development may support long-term prediction in dynamic sporting environments.

Finally, while quantitative measures such as RT provide objective performance information, effective TID decision-making also requires expert qualitative judgment (i.e., the “coach's eye”) (43, 44). The Fukuoka L-TID programme employs the “Shippo-tori (Tail-tag) game”, which illustrates this approach by assessing movement quality and strategic decision-making in dynamic contexts. This aligns with the concept of neuromuscular control as a complex skill dependent on the integration of sensory input and reflexive motor responses within dynamic actions (41). Supporting this qualitative focus, Yamaguchi et al. (48) reported that superior performance in the Shippo-tori game was the most prevalent selection feature among athletes who later attained elite status. By integrating maturity-independent quantitative indicators such as RT with expert qualitative assessments of movement patterns and tactical behaviour, TID decision-making may be strengthened and better aligned with multidimensional assessment frameworks (2). From a practical perspective, these findings suggest that neurocognitive training interventions, including reactive agility tasks, dual-task protocols, and sport-sampling activities designed to challenge perceptual-motor integration, are likely to yield the maximal developmental returns when implemented during the pre- and early-pubertal phases, prior to the onset of rapid somatic growth. As the primary window of neural development narrows and RT performance reaches a developmental plateau, the capacity for meaningful neurocognitive gains may diminish considerably. While this study did not test specific interventions, the relative stability of RT performance prior to PHV presents a potential window for early neurocognitive focus in TID pathways. Future research should evaluate the efficacy of such approaches in enhancing long-term athletic potential.

### 4.4 Limitations

Several limitations of the present study should be noted. First, the regional specificity of the Fukuoka L-TID cohort may limit direct generalisability. However, this study provides valuable longitudinal data on Asian athletes, a population that remains underrepresented in the broader sports science literature (20). Relatedly, a notable limitation involves the stringent inclusion criterion of complete longitudinal records, which resulted in an attrition rate of 74.9% (*n* = 96 of 383 graduates). Participants excluded due to missing data may differ systematically from those retained (e.g., due to injury, dropout, or varying levels of motivation), potentially introducing selection bias. Consequently, the retained cohort may represent a more physically resilient or committed sub-group, and these findings should be interpreted with this context in mind. Furthermore, the analysis did not account for individual injury history, cumulative workload, or sport-specific background, all of which significantly influence physical developmental trajectories during puberty. Second, imbalances in subgroup sizes, particularly the small number of late-maturing females (*n* = 7), likely reduced statistical power (45) to detect subtle maturation-sensitive interactions in attributes such as speed and power (22, 23). Consequently, non-significant findings for some outcomes should be interpreted cautiously. To mitigate this concern and ensure a more robust analysis of ranking stability, Kendall's *τ* was employed alongside Pearson's *r*. Kendall's *τ* is well-recognised for its superior performance with small samples and its robustness to outliers, providing a more conservative and reliable assessment of individual ranking trajectories than traditional parametric methods. Third, identifying PHV based on annual height measurements is retrospective and may underestimate instantaneous peak growth compared with more frequent monitoring (9, 40). Although annual measurement intervals represent standard practice in large-scale longitudinal research, this frequency limits real-time detection of growth spurt onset. To enhance the precision of maturity-informed programming, future programmes should consider incorporating quarterly height assessments, which would provide a more granular and prospective view of individual developmental trajectories. A related limitation concerns the maturity-based categorisation employed in this study, which was derived from Japanese general population norms (30, 31) given the current absence of validated Japanese athlete-specific PHV reference values. As athletes may exhibit systematically earlier or later PHV timing relative to the general population, this approach carries a risk of classification bias that may have influenced group assignments. Future studies should therefore prioritise the development of athlete-specific normative data for Japanese youth populations to refine maturity-based categorisation in TID contexts. Fourth, while the lack of specific intraclass correlation coefficients (ICCs) due to the retrospective design is a limitation, adherence to standardised national protocols and automated timing systems likely minimised measurement error. Fifth, the analysis did not control for environmental and behavioural factors (e.g., nutrition, sleep hygiene, and cumulative workload), which can influence growth tempo and performance development (11, 46, 47). Additionally, this study focused on general physical fitness rather than sport-specific skill acquisition, which remains an important research topic as athletes transition into specialised training (5). Finally, as our monitoring ended at G9, we cannot rule out the possibility that early measurements (e.g., G4) may still hold long-term predictive value for adult performance once motor coordination stabilises post-PHV. Nevertheless, these findings offer a rare longitudinal perspective on talented Japanese youth, and provide practical value for refining regional/local TID frameworks.

### 4.5 Practical recommendations

To optimise the Japanese L-TID framework and similar programmes, we propose the following recommendations based on the present findings:

1. Implement maturity-informed assessments: Adopt evaluation criteria based on biological age rather than chronological age to avoid overvaluing early-maturing males, whose temporary advantages in sprint speed and explosive power often obscure long-term potential.
2. Increase monitoring frequency and optimise timing: Transition from annual to quarterly height assessments to accurately capture non-linear patterns of physical development. Practitioners need to recognise that assessments conducted around PHV are more susceptible to individual ranking fluctuations due to “adolescent awkwardness”. Consequently, long-term tracking and a sufficient adaptation period are essential before making high-stakes selection decisions.
3. Provide targeted post-PHV support for females: Practitioners should interpret post-PHV declines in weight-bearing endurance as a transient physiological constraint (driven by increased metabolic cost) rather than a loss of athletic talent. They should provide individualised workload adjustments and psychological support to assist female athletes in managing the combined demands of neuromuscular re-integration and increased body mass.
4. Prioritise early neuromuscular development: In alignment with the YPD model, use the pre-pubertal period as a “critical window” to establish foundational motor coordination. Creating this stable base is vital before the rapid anthropometric changes of the growth spurt occur.
5. Integrate multidimensional assessments: Combine maturity-independent quantitative data (e.g., RT) with qualitative expert evaluation (e.g., the tail tag game) to provide a more robust assessment of an athlete's movement intelligence, particularly during periods of physical performance volatility.

## Conclusion

This long-term longitudinal investigation reveals that biological maturation influences physical fitness in a sex-specific manner: somatic growth appears to accelerate sprint speed and explosive power in males, while presenting a transient constraint for weight-bearing endurance in females after PHV. In contrast, neurocognitive development improved consistently across both sexes and maturity groups, supporting the notion that some neural-related attributes (e.g., RT) develop relatively independently of somatic growth and may offer valuable indicators during early-stage TID. Crucially, proximity to PHV was associated with temporary instability in individual performance rankings, consistent with adolescent awkwardness and insufficient time for neuromuscular re-integration. Consequently, athletic potential should be interpreted with caution during the growth spurt. These findings suggest that TID programmes may benefit from emphasising early neurocognitive development and providing adequate post-PHV adaptation periods to distinguish maturity-related fluctuations from sustainable potential. By helping address the scarcity of longitudinal evidence in Japanese youth athletes, this study supports maturity-informed, multidimensional approaches to optimise athlete development systems in Japan and beyond.

## Data Availability

The datasets presented in this article are not readily available because data sharing is restricted due to ethical reasons and the terms of the approval granted by the first author's Institute Ethics Committee. The approval stipulated that data would be strictly managed and not shared with third parties. Requests to access the datasets should be directed to Masahiro Hagiwara, masahiro.hagiwara@jpnsport.go.jp.
